# Evaluation of the Integration of Genetics and Genomics Into Nursing Practice

**DOI:** 10.1111/jnu.70056

**Published:** 2025-11-23

**Authors:** Kathleen Calzone, Liz Stokes, Cheryl Peterson, Laura M. Yee, David Liewehr, Laurie Badzek

**Affiliations:** ^1^ Genetics Branch Center for Cancer Research, National Cancer Institute, National Institutes of Health, Sigma Theta Tau Chapter: Xi Bethesda Maryland USA; ^2^ Center for Ethics and Human Rights, American Nurses Association Silver Spring Maryland USA; ^3^ Nursing Programs, American Nurses Association, STTI Chapter: Beta Iota, Tau Silver Spring Maryland USA; ^4^ Office of Collaborative Biostatistics, Center for Cancer Research, National Cancer Institute, National Institutes of Health Bethesda Maryland USA; ^5^ Ross and Carol Nese College of Nursing, Penn State University, Sigma Theta Tau Chapters: Beta Sigma, Alpha Rho USA

**Keywords:** attitudes, competency, genetics, genomics, knowledge, nursing, skills

## Abstract

**Purpose:**

Assess US registered nurse genomic competency.

**Design:**

Administered the Genetics and Genomics Nursing Practice Survey (GGNPS).

**Methods:**

GGNPS assesses genomic knowledge, skills, attitudes, confidence, and utilization in nursing practice. Distributed by the American Nurses Association via email and online to US registered nurses. Results are analyzed using descriptive statistics and compared to 2010 data.

**Results:**

1065 registered nurses responded. Most (41%) were Master's prepared, actively seeing patients (51%) and 66% considered it very important to learn more about genomics. Most (55%) reported their genomic knowledge was poor yet 51% reported a patient initiated a genetic discussion with them in the past 3 months. 66% completed all knowledge score items with a median score of 9/12, no change from 2010. Only 26% had heard of the Essential Competencies. Most reported no genomic curricular content (64%); had not attended a genomic course since licensure (64%); intended to learn more about genomics (70%); and would attend a course on their own time (79%).

**Conclusions:**

Nurses felt genomics was important but have capacity deficits. Despite genomic discoveries and evidence‐based practice guidelines that impact healthcare quality and safety, 20 years after the Genomic Competencies were established (2005) nursing genomic practice capacity remains low.

**Clinical Relevance:**

Genomics is critical to the safe, quality nursing practice regardless of the level of academic training, clinical role, or specialty.

## 1 Introduction

Genomics is the entire set of genetic instructions found in a cell, including their interactions with each other, the environment, and the influence of other psychosocial and cultural factors (Calzone et al. 2024). Genomics can increase therapeutic efficacy, safety, quality, and reduce healthcare costs. Precision health and precision medicine are underpinned by genomics while considering individual lifestyle, behaviors and environmental contexts that account for each individual's unique attributes that contribute to their health (Calzone, Jenkins, et al. 2013). Genomics and precision health are clinically relevant throughout the entire healthcare continuum from before birth to after death. Therefore, both have implications for the entire nursing profession regardless of level of academic training, role, or clinical specialty. Ongoing work with the American Nurses Association (ANA) has focused on policy efforts aimed at accelerating precision health and genomics knowledge, skills, and integration into nursing practice to improve health outcomes. This work included updating the 2009 Essential Genetic and Genomic Nursing Competencies and Outcome Indicators as well as establishing precision health nursing competencies (Calzone et al. 2024).

Critical to informing the next steps of the ANA initiative is to assess nursing knowledge, skills, attitudes, and practices in genomics to determine what progress has been made since the last assessment conducted from October 2009 to January 2010 (Calzone, Jenkins, et al. 2013). The instruments used for this study include the validated Genetic and Genomic Nursing Practice Survey (GGNPS) (Calzone et al. 2016; Plavskin et al. 2023) as well as the newly developed Precision Health Nurses' Capacity Scale which has undergone face and content validity (Fangonil‐Gagalang et al. 2024). The instruments were combined to reflect a single online survey for distribution to the nursing community. Data from the GGNPS is being compared with prior data collected from ANA following the development of the initial Essential Genetic and Genomic Nursing Competencies (Calzone, Jenkins, et al. 2013). Data from this study will benchmark any genomic capacity changes since the onset of the genomic competencies. These data will provide the necessary information to design and then measure the effectiveness of any nursing educational initiatives as a result of the findings.

## 2 Background

The largest hurdles to optimizing evidence‐based utilization of genomics are healthcare provider capacity, which is limited across all healthcare disciplines. For example, the extent of existing genetic tests is extraordinary. As of March 28, 2025, the Genetic Testing Registry reports 68,303 tests for 18,703 genes, performed in 397 laboratories, for 11,076 conditions Testing types can include germline pharmacogenomics, somatic and/or germline single site, multi‐gene panels and whole exome or genome tests and can be either clinical or direct‐to‐consumer tests (Centers for Disease Control and Prevention 2024). Genomic tests underpin precision health which builds upon the genomic foundations adding critical health factors such as personal behaviors and environmental influences (Fangonil‐Gagalang et al. 2024).

Taken together, precision health and genomics exemplify a complex competency for the following reasons: not all interventions are observable; for example, pharmacogenomic tests, when used appropriately can optimize medication selection while minimizing adverse drug reactions; the complexity of the language and science of genomics and precision health; the speed at which evidence is generated that is applicable to clinical practice; and many providers have limited or even no foundation in genomics let alone precision health (Calzone, Badzek, et al. 2013). These complexities slow adoption rates especially in economically, ethnically, and racially disadvantaged communities and the strategies used for implementation may influence the rate of adoption (Fontaine et al. 2024; Rogers 2003).

The features of genomic interventions also play a role. To illustrate, many genomic interventions are not observable, such as the use of a pharmacogenomic test to inform medication selection and dosing resulting in the expected medication response with minimal toxicities. Therefore, to adopt pharmacogenomics requires sufficient underpinning on how these tests can inform prescribing and improve outcomes. Yet many nurses (48%) with prescriptive privileges reported not even knowing what pharmacogenomic test to order and most (84%) never used pharmacogenomic evidence‐based guidelines (Fulton et al. 2024). These competency deficits are only expanded with the extension of genomics into an even broader environment of precision health. Nursing, as the most trusted healthcare provider, is underpinned by upholding the quality and safety of healthcare. Genomics, when used correctly, has been proven to improve the quality and safety of healthcare. Therefore, nursing has a clinical, moral, and ethical obligation to establish a multi‐faceted initiative to overcome educational, organizational, and nursing practice deficits in genomics and precision health. As part of an interprofessional team, genomics and nursing practice have been found to improve health outcomes (Peterson et al. 2024).

## 3 Study Justification

The initial Genetic and Genomic Nursing Competencies were established with the support from the American Nurses Association in 2006 (Jenkins and Calzone 2007). This was followed by an expansion of those competencies to establish outcome indicators consisting of specific areas of knowledge needed to achieve each competency and the corresponding clinical performance indicators (Calzone et al. 2011). Since then, the clinical utility and application of genomics have expanded into all disease, risk, and wellness entities and have been integrated into evidence‐based practice standards such as cancer guidelines established by the National Comprehensive Cancer Network (Network 2024) and pharmacogenomic guidelines such as those established by the Clinical Pharmacogenomic Implementation Consortium (Guidelines 2023). Despite a series of implementation initiatives since the competencies were established (Calzone et al. 2014; Jenkins and Calzone 2014), there continues to be evidence that nursing competency in genomics remains limited which is impacting the quality and safety of nursing care (Fulton et al. 2024). With the latest version of the genomic nursing competencies updated (Calzone et al. 2024) and the expansion of genomics into Precision Health, reassessing the current capacity of the nursing workforce in Precision Health and Genomics (PH&G) would inform capacity and potential deficits that require remediation. This study reports specifically on the genomic aspects of the assessment.

## 4 Aims

The primary aim of this study was to assess registered nurse attitudes, receptivity, knowledge, confidence, and utilization of genomics in practice using the Genetics and Genomics Nursing Practice Survey (GGNPS) instrument. The secondary aim was to compare these data with similar data collected from October 2009 to January 2010 (Calzone, Jenkins, et al. 2013) using the GGNPS and the same recruitment strategy. This study also collected information on Precision Health variables that are reported separately.

## 5 Methods

### 5.1 Theoretical Framework

The Diffusion of Innovation (Rogers 2003) theoretical framework guided this study. This conceptual framework provides the guidance to consider characteristics of the innovation such as complexity, advantages, observability, trialability, and the social system which in this case is the healthcare community. Additionally, this framework considers factors that influence adoption, such as the attitudes and knowledge of genomics held by the adopters—in this case nurses. All these variables can influence adoption and impact the diffusion of the innovation which in this case is genomics (Calzone, Jenkins, et al. 2013).

### 5.2 Instrument

The GGNPS was the instrument utilized in this study. The GGNPS measures the following Diffusion of Innovation (Rogers 2003) domains: attitudes and receptivity; knowledge and competency; confidence, social system, and utilization of genomics in practice. This instrument has been refined over time based on instrument validation assessments (Calzone et al. 2016; Plavskin et al. 2019, 2023). Instrument items are associated with family history and the genomics of common diseases and therefore are not dependent on cost or access to genetic testing. The items are consistent with the competencies defined in the 3rd Edition of the Essentials of Genomic Nursing: Competencies and Outcome Indicators (Calzone et al. 2024) for all nurses regardless of role, specialty, or level of academic training. Item formats include multiple choice, yes/no, and Likert scale questions. Additionally, some GGNPS questions were refined. Prior versions of the GGNPS confidence question response options consisted of a 5‐item Likert scale with a middle anchor. The confidence response options were revised to be a dichotomous response option of confident versus not at all confident. Otherwise, the primary difference between this instrument and the prior assessment was that this instrument was expanded to include a new section on Precision Health. Those items are not reported in this manuscript which is limited to the Genomic items. Precision Health Outcomes have been reported separately (Fangonil‐Gagalang et al. 2024). The survey was administered online using Survey Monkey and was estimated to take 20 min to complete.

### 5.3 Recruitment

The resources of the American Nurses Association (ANA) were used to recruit for this study. This included an email to all ANA members, posting on nursingworld.org, email announcements to subscribers of the ANA daily SmartBrief, and the weekly Nursing Insider e‐newsletter. The genetic nursing competency listserv maintained at the National Cancer Institute was also utilized for dissemination. The survey was available to complete for six weeks from June 1, 2023 to July 14, 2023.

### 5.4 Eligibility

Participants had to self‐report being a practical, vocational, or registered nurse prepared at any level of education (diploma to doctorate) to participate in the study. There was no incentive provided for participation.

### 5.5 Regulatory Approval

This study was reviewed and determined not to be human subjects research as the data generated was for quality improvement purposes and therefore did not meet the definition of research. Additionally, the study was considered Code of Federal Regulations (45 CFR 46) exempt because data were collected without any direct interaction with participants, no personally identifiable information was collected, and participants could skip any question they did not wish to answer so there were no risks to participation (U.S. Department of Health and Human Services 2023).

## 6 Data Analysis

Results were analyzed using descriptive statistics. Comparison of the current survey findings was compared to the prior National Nursing Workforce Survey using the GGNPS in which data was collected from October 2009 to January 2010 (Calzone, Jenkins, et al. 2013). The purpose of that comparison was to look for differences in the population and overall knowledge differences between the two separate studies which used the GGNPS and the same recruitment strategy. Importantly, the GGNPS has undergone refinement as part of the ongoing instrument validation since that initial study. Therefore, only questions that were identical were compared. The survey represented a random sample; therefore a descriptive approach including effect sizes and corresponding 95% and 99% confidence intervals was utilized to compare the 2023 data to the data collected in 2010.

## 7 Results

### 7.1 Population

The 2023 survey had 1066 respondents. Of those, one respondent entered the survey but did not complete any items and was excluded from the analysis leaving a total of 1065 included in the analysis. Of those that completed the demographic questions (Table 1), most nurses were older, mean 58 years of age and highly educated with 41% reporting their highest nursing degree was a master's and 24% a doctorate. This differs from the 2010 survey in which the cohort was younger, mean 51 years of age, and most were prepared at the baccalaureate (41%) or master's (31%) levels (Calzone, Jenkins, et al. 2013). There also are differences in primary areas of practice. In the current survey, most were educators (22%) followed by staff nurses (20%). In 2010, most respondents were staff nurses (54%) with the rate of educators (20%) being similar (Calzone, Jenkins, et al. 2013). However, changes in the 2023 instrument reflect collection of practice roles not collected in 2010 inclusive of clinical roles such as nurse practitioners (17%). Despite that, the overall time nurses reported seeing patients was lower in 2023 (42%) compared to 2010 (54%) (Calzone, Jenkins, et al. 2013).

**TABLE 1:** Population demographics.

Demographic variables	2010 Survey, *N* = 619 (Calzone, Jenkins, et al. 2013)	2023 Survey, *N* = 1066
Age	Mean 50.7, range 21–76	Mean 58.3, range 24–83
*Highest degree*	*N* = 483, missing 136	*N* = 633, missing 433
Diploma	*N* = 16, 3.3%	*N* = 10, 1.6%
Associate degree	*N* = 83, 17.2%	*N* = 50, 7.9%
Baccalaureate degree	*N* = 196, 40.6%	*N* = 161, 25.4%
Master's degree	*N* = 148, 30.6%	*N* = 260, 41.1%
Doctorate degree	*N* = 39, 8.1%	*N* = 151, 23.9%
*Number of year worked in nursing*		
Years	Not asked	*N* = 627, missing 439 Mean 29.39, range 1–50
*Primary area of practice*	*N* = 427, missing 192	*N* = 631, missing 435
Staff nurse	*N* = 231, 54.1%	*N* = 127, 20.1%
Head nurse	*N* = 38, 8.9%	*N* = 19, 3.0%
Nurse practitioner		*N* = 105, 16.6%
Clinical nurse specialist		*N* = 22, 3.5%
Educator	*N* = 85, 19.9%	*N* = 136, 21.6%
Supervisor		*N* = 16, 2.5%
Director/Assistant director		*N* = 50, 7.9%
Researcher	*N* = 16, 3.7%	*N* = 21, 3.3%
Student	*N* = 9, 2.1%	
Consultant		*N* = 26, 4.1%
Case manager		*N* = 19, 3.0%
Other	*N* = 48, 11.2%	*N* = 90, 14.3%
*Percent of time seeing patients*	*N* = 427, missing 192	*N* = 607, missing 459
	Mean 54.1%, range 0–100	Mean 41.6%, range 0–100

### 7.2 Attitudes/Receptivity

Most respondents indicated that it was important (*N* = 705, 66%) or very important (*N* = 300, 28%) for nurses to become more educated about the genetics of common diseases. These data are largely unchanged from 2010 in which respondents reported it was important (*N* = 410, 68%) or very important (*N* = 162, 27%) to become more educated in the genomics of common disease (Calzone, Jenkins, et al. 2013). As summarized in Table 2, the top three advantages of genomic practice integration included: better decisions about recommendations for preventative services (*N* = 1023, 96%); better treatment decisions (*N* = 993, 94%); and improved services to patients (*N* = 972, 92%). The data from 2010 showed slightly lower opinions on the advantages: better decisions about recommendations for preventative services (*N* = 525, 85%); better treatment decisions (*N* = 490, 79%); and improved services to patients (*N* = 427, 69%) (Calzone, Jenkins, et al. 2013). The top three disadvantages of genomic practice integration included: would increase insurance discrimination (*N* = 851, 81%); not reimbursable/too costly (*N* = 792, 76%); and increase patient anxiety about risk (*N* = 608, 58%). Data from 2010 found slightly lower reported disadvantages compared to the current study: would increase insurance discrimination (*N* = 386, 62%); and increase patient anxiety about risk (*N* = 290, 47%) (Calzone, Jenkins, et al. 2013).

**TABLE 2:** Advantages/Disadvantages.

Outcomes of practice integration	Advantages	
	Advantage^a^	No advantage^a^
Better decisions about recommendations for preventive services	*N* = 1023 (96%)	*N* = 39 (4%)
Better treatment decisions (e.g., which drugs to prescribe)	*N* = 993 (94%)	*N* = 68 (6%)
Improved services to patients	*N* = 972 (92%)	*N* = 84 (8%)
Better adherence to clinical recommendations	*N* = 887 (84%)	*N* = 171 (16%)
Genomic risk triaging could make better use of visit time	*N* = 868 (82%)	*N* = 188 (18%)

Outcomes of practice integration	Disadvantages	
	Disadvantage	No disadvantage
Would take too much time	*N* = 438 (42%)	*N* = 594 (56%)
Not reimbursable/Too costly	*N* = 792 (76%)	*N* = 244 (24%)
Need to “re‐tool” professionally	*N* = 396 (38%)	*N* = 633 (62%)
Increse patient anxiety about risk	*N* = 608 (58%)	N‐435 (42%)
Would increase insurance discrimination	*N* = 851 (81%)	*N* = 199 (19%)

^a^ Percents rounded to the nearest whole number.

### 7.3 Confidence

The current survey (Table 3) found that only a small proportion of nurses were confident (*N* = 301, 33%) in their ability to decide what family history information was needed to inform a patient's susceptibility to common diseases. This represents only a slight improvement from the 2010 assessment (*N* = 153, 27.5%) (Calzone, Jenkins, et al. 2013). Similarly, there was a modest lowering of confidence in giving patients information on the risk (2010‐*N* = 338, 61%; 2023‐*N* = 419, 46%) and limitations (2010‐*N* = 328, 60%; 2023‐*N* = 424, 46%) of genetic testing for common diseases. Interestingly, that was not found for giving patients information on the benefits of genetic testing for common disease with fewer nurses in 2010 (*N* = 279, 51%) reporting a lack of confidence compared to the 2023 data (*N* = 561, 62%). Lastly, slightly fewer nurses in the current survey (2010‐*N* = 304, 55%; 2023‐N = 424, 47%) reported a lack of confidence in facilitating referrals for genetic services (Calzone, Jenkins, et al. 2013).

**TABLE 3:** Confidence.

Confidence in doing the following	Confident^a^	Not at all confident^a^
Decide what family history informaiton is needed to tell something about a patient's genetic susceptibility to common diseases	*N* = 301 (33%)	*N* = 616 (67%)
Discuss how family history affects recommended screening intervals	*N* = 250 (27%)	*N* = 669 (74%)
Decide which patient would benefit from a referral for genetic counseling and possible testing for susceptibility to common diseases	*N* = 433 (47%)	*N* = 485 (53%)
Access reliable and current informaiton about genetics and common diseases	*N* = 355 (39%)	*N* = 560 (61%)
Give patients information about the *risks* of genetic testing for common diseases	*N* = 497 (54%)	*N* = 419 (46%)
Give patients information about the *benefits* of genetic testing for common diseases	*N* = 351 (39%)	*N* = 561 (62%)
Give patients information about the *limitations* of genetic testing for common diseases	*N* = 490 (54%)	*N* = 424 (46%)
Facilitate referrals for genetics services for common diseases	*N* = 486 (53%)	*N* = 424 (47%)

^a^ Percents rounded to the nearest whole number.

### 7.4 Knowledge/Competency

**FIGURE 1:**
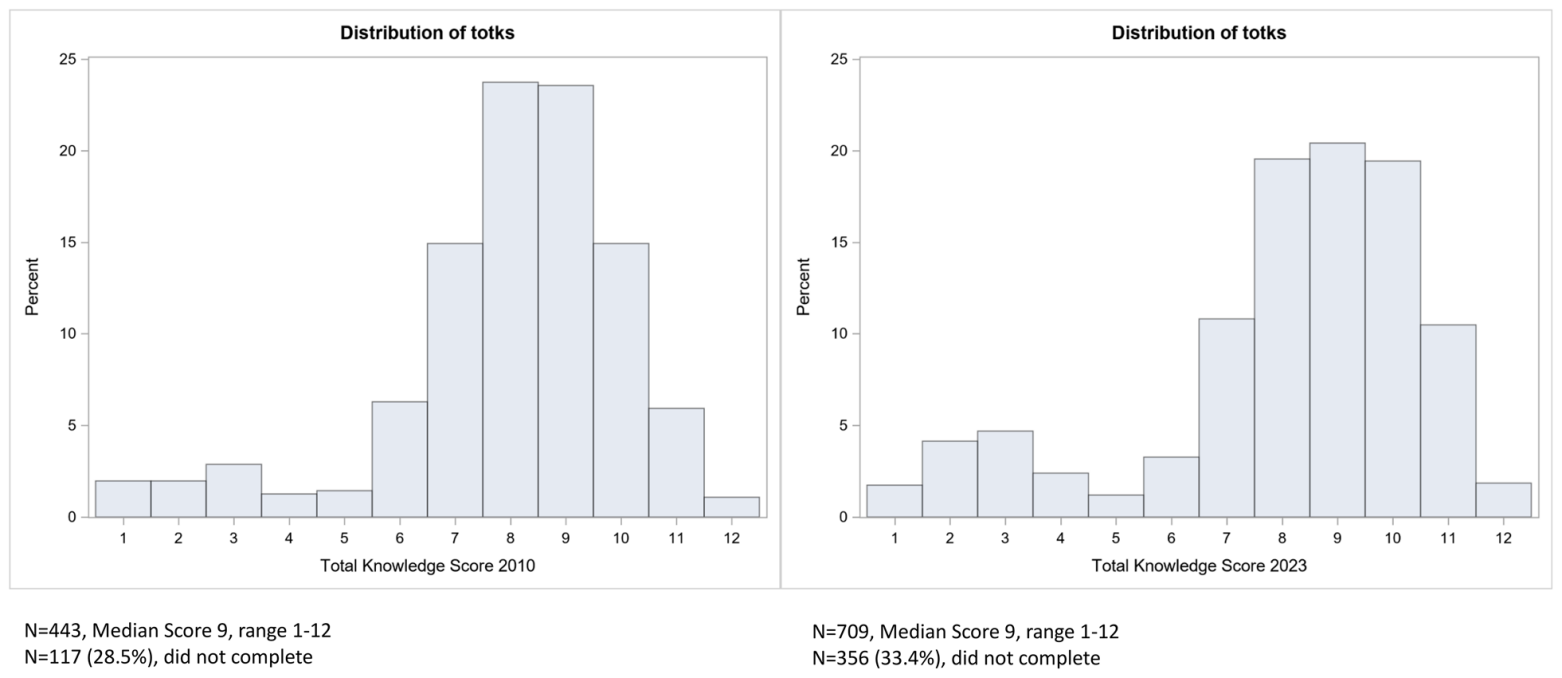
Knowledge score histograms, 2010 compared to 2023.

Overall, 709 participants (67%) completed all 12 knowledge score items (Table 4). The median knowledge score was 9/12, with a range of 1–12. The total knowledge score represents no change from the data collected in 2010 (Calzone, Jenkins, et al. 2013). Knowledge score histograms illustrated in Figure 1 document subtle differences between the 2010 and 2023 knowledge scores. This includes slightly more individuals in 2023 with low scores in the 2–3 range and more nurses in 2010 scoring 8 and 9 compared to the current 2023 cohort. Additionally, the number of nurses not completing the knowledge score items increased slightly from 28.5% in 2010 to 33.4% in 2023.

**TABLE 4:** Knowledge score items.

Item number	Item	Correct *N* (%)^a^	Incorrect *N* (%)^a^
1	A family history that includes only first‐degree relatives such as parents, siblings and children should be taken on every new patient	*N* = 192 (27%)	*N* = 517 (73%)
2	A family history that includes second and third‐degree relatives such as grandparents, aunts, uncles, and cousins should be taken on every new patient	*N* = 492 (69%)	*N* = 217 (31%)
3	Family history taking should be a key component of nursing care	*N* = 648 (91%)	*N* = 61 (9%)
4	There is a role for nurses in counseling patients about genetic risks	*N* = 603 (85%)	*N* = 106 (15%)
5	Do you think that genetic risks (e.g., as indicated by family history) has clinical relevance for breast cancer	*N* = 705 (99%)	*N* = 4 (1%)
6	Do you think that genetic risks (e.g., as indicated by family history) has clinical relevance for colon cancer	*N* = 704 (99%)	*N* = 5 (1%)
7	Do you think that genetic risks (e.g., as indicated by family history) has clinical relevance for coronary heart disease	*N* = 705 (99%)	*N* = 4 (1%)
8	Do you think that genetic risks (e.g., as indicated by family history) has clinical relevance for diabetes	*N* = 704 (99%)	*N* = 5 (1%)
9	Do you think that genetic risks (e.g., as indicated by family history) has clinical relevance for ovarian cancer	*N* = 706 (99.6%)	*N* = 3 (0.4%)
10	Extent to which family history supports clinical decsions (such as administering drugs prescribed)	*N* = 378 (53%)	*N* = 331 (47%)
11	The DNA of sequences of two randomly selected healthy individuals of the same sex are 90%–95% identical	*N* = 208 (29%)	*N* = 501 (71%)
12	Most common diseases such as diabetes and heart disease are caused by a single gene variant	*N* = 273 (39%)	*N* = 436 (61%)

^a^ Percents rounded to the nearest whole number unless unable to accurately reflect responses to an item.

Median 2023 knowledge scores varied based on level of academic preparation with lower levels of education having lower median knowledge scores and increasing scores as the level of education increased. Specifically, Diploma prepared nurses had a median score of 8, Associate or Baccalaureate degree nurses 8.5, Master's degree 9, and Doctorate degree 10. Role also showed differences in knowledge scores with staff nurses and head nurses both having a median of 8, and higher scores in researchers and clinical nurse specialists, median 10.

Assessing specific knowledge items (Table 4) reveals some areas where most nurses are knowledgeable, such as whether family history has clinical relevance for different diseases such as breast, ovarian, and colon cancer, coronary heart disease, and diabetes in which 99% or more answered correctly. However, findings also identify some knowledge deficits such as the scope of family history assessment in which 73% answered incorrectly that a family history that includes only first‐degree relatives such as parents, siblings and children should be taken on every new patient. These findings represent no change from the 2010 data in which the incorrect percent was 72.9%. Additionally, basic genetic concepts responses also documented knowledge deficits. In a 2023 study 71% incorrectly answered that the DNA sequences of two randomly selected health individuals of the same sex are 90%–95% identical (Calzone, Jenkins, et al. 2013). This finding is slightly worse than 2010 in which 64.3% incorrectly answered this question. In the knowledge statement that most common diseases such as diabetes and heart disease are caused by a single gene variant the data are largely unchanged with most answering incorrectly (2010–60.6% and 2023–61%).

### 7.5 Social System

The environment in which a nurse practices is fundamental to both encouraging and enabling education in areas of rapidly changing practice and documented knowledge deficits of which genomics applies to both. Most nurses (*N* = 527, 70%) indicated they intend to learn more about genetics. However, only about half (*N* = 355, 48%) know whether they could attend a course during work hours. Most (*N* = 590, 79%) indicate they would attend a course on their own time. Unfortunately, the majority (*N* = 390, 42%) of nurses in the current survey reported that their senior staff do not see genetics as an important part of their role or do not know the views on the importance placed on genetics of their senior staff (*N* = 283, 38%). Similarly, most (*N* = 312, 42%) indicated that they do not know or responded that senior staff do not see genomics as an important part of their role (*N* = 290, 39%). These data are similar to data from 2010 in which most nurses (73%) indicated they were willing to attend a genetics course on their own time, but did not know (22%) whether they would be able to attend a genetics course during work hours (Calzone, Jenkins, et al. 2013).

### 7.6 Genomics Utilization in Practice

The assessment of genetics and genomics use in practice (Table 5) included a focus on family history that is not cost or technology dependent and is in the domain of all nurses. Of those that collected family history information, less than half (34.8%) always collected one of the essential indicators of inherited predisposition to disease, the age in which the condition was diagnosed. Additionally, only half of nurses (50.7%) always collected both maternal and paternal lineages.

**TABLE 5:** Utilization of genomics in practice.

Item	Always *N* (%)	Sometimes *N* (%)	Never *N* (%)	Missing *N* (%)
*Family history information collected*				
Age at diagnosis of condition	370 (34.8%)	280 (26.3%)	117 (11.0%)	297 (27.9%)
Relationship to the patient	672 (63.2%)	62 (5.8%)	34 (3.2%)	296 (27.8%)
Race/Ethnic background	368 (34.6%)	223 (21.0%)	170 (16.0%)	303 (28.5%)
Age of death from condition	419 (39.4%)	234 (22.0%)	111 (10.4%)	300 (28.2%)
Both sides of the family	539 (50.7%)	179 (16.8%)	46 (4.3%)	300 (28.2%)

Item	Essential *N* (%)	Don't know *N* (%)	Not at all *N* (%)	Missing *N* (%)
*Support clinical decisions (such as administering drugs prescribed)*				
Genetic test result	405 (38.1%)	294 (27.6%)	51 (4.8%)	314 (29.5%)
Family history	578 (54.3%)	152 (14.3%)	45 (4.2%)	289 (27.2%)

Item	Frequently *N* (%)	Occaision‐ally *N* (%)	Rarely *N* (%)	Never *N* (%)
*Support general clinical decisions*				
Used family history to facillitate clinical decisions	130 (16.7%)	224 (28.7%)	116 (14.9%)	310 (39.5%)
Facilitated referrals to genetic services	27 (3.5%)	72 (9.2%)	95 (12.2%)	586 (75.1%)

The utilization of genetics and genomics practice (Table 5) included a focus on family history which involves no cost, is not technology dependent, and is in the domain of all nurses. Of those that collected family history information, less than half (34.8%) always collected one of the essential indicators of inherited predisposition to disease, the age at which the condition was diagnosed. Additionally, only half of nurses (50.7%) always collected both maternal and paternal lineages. In the domain of medication administration (Table 5), most do not use genomic test results (38%) or family history (54%) to support the administration of drugs prescribed. The use of family history to support general clinical decisions (Table 5) found that most (*N* = 310, 39.5%) never used family history to facilitate a clinical decision. However, many nurses reported occasionally (*N* = 224, 28.7%) or frequently (*N* = 130, 16.7%) using family history to facilitate clinical decisions. Despite this, most (*N* = 586, 75.1%) never facilitated a referral to genetic services.

## 8 Limitations

The demographic variables collected in the current study are more extensive than the 2010 study because of modifications to the GGNPS. Therefore, demographic variables do not completely align limiting some of the ability to compare with the prior National Nursing Workforce study (Calzone, Jenkins, et al. 2013). To help address this limitation, we assessed how survey respondents compared to the national nursing workforce demographics by comparing our data to the latest National Council of State Boards of Nursing (NCSBN) national nursing workforce study (Smiley et al. 2025). The NCSBN data, collected in 2024, found that most nurses were age ≥ 65 (18.4%) followed by 60–64 (11.6%). In comparison, this study found most nurses were slightly younger, with a mean respondent age of 58.3. The NCSBN study also reported that most nurses held a baccalaureate degree (47.8%) or an associate degree (22.8%). In contrast, this study consisted of mostly nurses with a master's degree (41.1%), followed by a doctoral degree (23.9%), with only 25.4% reporting a baccalaureate degree and 7.9% an associate degree. There is no direct comparison with the NCSBN data and this study on the number of years worked in nursing. However, comparing data from NCSBN on the number of years an individual was licensed as a nurse is a close corollary. NCSBN data found that most nurses reported licensure for 0–10 years (32.1%) followed by 11–20 years (24.9%). This study found that most reported a mean of 29.4 years in practice (range 1–50) as a nurse indicating this study had a slightly more experienced cohort. The age of the nurses in this study appears to be slightly younger; nurses participating in the 2023 study had a higher level of academic education and slightly more years of experience. Based on this comparison, the nurses in this study do not completely represent the United States national nursing workforce as of 2024. Lastly, this study cannot calculate an overall response rate given the method of recruitment.

An additional limitation of this work is that the GGNPS instrument was not developed to detect differences over time despite the instrument being well validated. While the authors appreciate that concern, the GGNPS has been used in multiple studies, and relevant to this possible limitation is the use of the GGNPS in the Method to Integrate a New Competency (MINC) into practice study. This was a pre/post genomic capacity building intervention study of 8150 registered nurses conducted at 23 American Nurses Credentialing Center designated Magnet intervention hospital nursing staff compared to 2 Magnet control hospital nursing staff. That study found the GGNPS could detect even subtle changes (Calzone et al. 2018).

## 9 Discussion

The participants in this study are more highly educated than the US national nursing workforce (Smiley et al. 2025). Additionally, when compared to the prior national nursing workforce study, 65% reported their highest degree is a Master's or Doctoral degree compared to 39% in 2010. Likely contributing to this change is the increase in nursing practitioners in the intervening years documented by data from the NCSBN which shows a significant rise from 2012, *N* = 105,780 compared to 2021, *N* = 234,690 (National Council of State Boards of Nursing 2023). With that increase in education and training, one could reasonably hypothesize that this would translate to more knowledge, skills, and abilities in genomics. However, that is not the case; the overall mean knowledge scores are unchanged, 9/12. The unchanged knowledge score is not surprising. Most nurses (*N* = 479, 64%) indicated that their nursing curriculum did not include genomic content. Additionally, most nurses (*N* = 478, 64%) responded that they had not attended any courses that included genomics since licensure. Additionally, data on the advantages and disadvantages point to areas in which nurses have a lack of understanding. For example, most nurses indicate that a disadvantage of integrating genomics into practice is because it is not reimbursable and too costly (76%), and it would increase insurance discrimination (81%). However, germline multigene panel tests, for example can be performed for more than 90 genes for approximately $250 depending on the laboratory; most insurers cover that cost, and clinical laboratories work with patients/families to facilitate coverage. Additionally, cases of insurance discrimination are limited and there is some legal protection provided by the Genetic Information Non‐Discrimination Act which was enacted in 2008 (“H.R.493‐Genetic Information Nondiscrimination Act of 2008” 2008). Both examples highlight areas that may be the result of nurses not being fully informed, which requires more detailed assessment and may be amenable to remedial education.

Achieving genomic competency is multi‐faceted and varies based on the role of the nurse, their level of academic preparation, practice specialty, and their clinical role. Despite efforts, nurses have not upskilled in genomics sufficiently to use genomics to improve the quality and safety of patient care. There are differences between clinical and academic nurse educators, yet both need to have the capacity to teach genomic concepts. Clinical nurses prepared at the Associate or Baccalaureate level have a scope of practice that differs from Advanced Practice Registered Nurses (APRN) who may have prescriptive privileges. Yet all registered nurses need to have competency in administering and/or managing medications and their outcomes. This includes the ability to recognize genomic phenotypes that impact medication outcomes including toxicities which should be reported to the ordering provider. Importantly, this does not require a genetic test but does require all nurses to have a sufficient underpinning in pharmacogenomics to recognize a phenotype and manage it appropriately based on the nurse's scope of practice. Most importantly, there is the pharmacogenomic capacity of APRNs with prescriptive privileges. A recent study found that most (84%) APRNs with prescriptive privileges do not use pharmacogenomic evidence‐based Clinical Pharmacogenetics Implementation Consortium (CPIC) guidelines (Clinical Pharmacogenetic Implementation Consortium: Guidelines 2024). Only 29% reported ordering a pharmacogenomic test in the past year with 48% indicating this was because they do not know what test to order. Importantly, pharmacogenomic curricular content did not influence pharmacogenomic familiarity, confidence, or test ordering (Fulton et al. 2024). This points to whether the curricular content is adequate to prepare nurses to utilize pharmacogenomics in practice. These findings are similar to those of physician providers. A recent scoping review found that while physician providers found pharmacogenomics of value to patient care, they also lacked sufficient knowledge to use pharmacogenomics in practice (Keeling et al. 2021). There is an extensive literature on models to facilitate the use of pharmacogenomics by prescribers including but not limited to institutional infrastructure and education initiatives (Shugg et al. 2024), pharmacist‐assisted programs (Arnall et al. 2019), and point‐of‐care decision support (Hoffman et al. 2016). This points to pharmacogenomics being an interprofessional challenge that may be amenable to a larger cooperative effort.

Additionally, the capacity of faculty to teach not just pharmacogenomic content but other genomic content as well warrants further assessment. Importantly, teaching capacity is not limited to the academic environment but extends to the clinical continuing environment given the evidence documenting a lack of genomic capacity in practicing nurses.

Genomics is especially complex because of the rapidly evolving evidence base. With the substantial reduction in costs of genomic analyses, the discovery of the genomic underpinnings of disease has led to the development of treatments that target the underlying genomics of the condition. Additionally, as the evidence continues to accumulate, pre‐emptive genetic testing, especially in the context of pharmacogenomics is being recommended to reduce toxicities and save costs by using genomics to select the optimal treatment for the individual patient that also minimizes toxicities (Haidar et al. 2022; Massmann et al. 2024; Pereira et al. 2024). Consequently, healthcare is actively transitioning into Precision Medicine and Precision Health corresponding to an expanded role for nurses (Al‐Kaiyat 2018; Clinton et al. 2020).

Genomics represents a clear quality and safety issue of nursing care that has persisted for years as the original genomic competencies were established in 2009, more than 15 years ago. This is not for lack of effort to facilitate genomic nursing competency in both academic (Jenkins and Calzone 2014), practice (Calzone et al. 2014), and research settings (Hickey et al. 2013; Regan et al. 2019). In nursing, what is most concerning is that the integration of evidence‐based genomics into practice continues to expand (Bowdin et al. 2016) yet the evidence continues to support that many nurses continue to have ongoing deficits that have not changed. This deficit persists despite the expansion of genomics and other omic technologies coupled with a reduction in prices and an expansion of the evidence‐based applications and guidelines permitting the routine use of these tests in common health decision‐making such as but not limited to cancer (Network 2024), cardiovascular disease (Musunuru et al. 2020), and medication selection/dosing (Clinical Pharmacogenetic Implementation Consortium: Guidelines 2024). While reported advantages of genomic practice integration slightly increased, confidence remained low for even the simplest cheapest genomic test, family history. However, there is some good news as the study found a slight increase in self‐reported ability to discuss genetics or facilitate referrals.

Overcoming this challenge is multi‐faceted. In the academic setting both guidelines for education and faculty training need to be addressed. The American Association of Colleges of Nursing Essentials provide degree‐level competencies and curricular content to guide academic programs. As AACN moves forward with transitioning to competency‐based education, it is essential to integrate genomics as it relates to individual and population health including disease risk, diagnosis, therapeutic decision‐making, and symptom management at all levels of training. Currently, genomics is only included at the graduate level in one item 2.2i which speaks to genetics, genomics, and pharmacogenetics as an example of individualized information relevant to personalized healthcare (American Association of Colleges of Nursing 2021). This includes providing sufficient training and support to faculty to teach this content. Similarly, nurses already in practice require continuing education in genomics. This is not limited to nurse practitioners but includes nurses at all levels of education and practice. This requires continuing nurse educators to have the capacity and resources to teach this content. Notably, teaching and learning resources are extensive and most are freely available from the National Human Genome Research Institute, the National Cancer Institute, and the Center for Disease Control and Prevention to name a few.

Participants were an older, more experienced, and higher educated cohort compared to the prior study which could be argued should have resulted in greater genomic capacity, which was not the case. Additionally, these findings occurred in the face of expanding high‐throughput omics technologies with an increasing evidence base of clinical applications that are no longer limited to genomics, but expanded to include transcriptomics, proteomics, metabolomics, epigenomics, and other well‐established omics. Technologies that are increasingly more accessible, affordable, and can be performed rapidly to inform healthcare decision‐making. Nurses, who typically spend the most time with the patient and their family, are often the first person to which questions about tests in general, including genomic tests are directed.

## 10 Conclusion

Findings from this study document that most nurses still report it is important for nurses to be educated about genomics. However, data indicate that while participant nurses report the advantages of using genomics are slightly increased, their capacity to use genomics in practice remains limited and largely unchanged in more than 10 years. Nurses at all levels of education and practice settings must increase their capacity in genomics and other omics to continue to provide safe and evidence‐based care. These data can be used to inform omic education targets leveled for nursing, nursing faculty, and nursing continuing education faculty to address this deficit.

## Conflicts of Interest

The authors declare no conflicts of interest.

## Clinical Resources

- Clinical Pharmacogenetic Implementation Consortium (CPIC) Guidelines. https://cpicpgx.org/guidelines/.
- Essentials of Genomic Nursing: Competencies and Outcome Indicators. https://www.nursingworld.org/nurses‐books/ana‐books/ebook‐essentials‐of‐genomic‐nursing‐competencies‐/.
- National Human Genome Research Institute About Genomics. https://www.genome.gov/about‐genomics.

## Data Availability

The data that support the findings of this study are available on request from the corresponding author. The data are not publicly available due to privacy or ethical restrictions.
